# Central intake to improve access to physiotherapy for children with complex needs: a mixed methods case report

**DOI:** 10.1186/s12913-016-1700-3

**Published:** 2016-08-31

**Authors:** Kristy D. M. Wittmeier, Gayle Restall, Kathy Mulder, Brenden Dufault, Marie Paterson, Matthew Thiessen, Lisa M. Lix

**Affiliations:** 1 George and Fay Yee Centre for Healthcare Innovation, 375-753 McDermot Avenue, Winnipeg, MB R3E 0T6 Canada; 2 Department of Pediatrics and Child Health, Rady Faculty of Health Sciences, Health Sciences Centre, University of Manitoba, CE208-840 Sherbrook St., Winnipeg, MB R3A 1S1 Canada; 3 Physiotherapy, Health Sciences Centre, CH246-840 Sherbrook St., Winnipeg, MB R3A 1S1 Canada; 4 Department of Occupational Therapy, College of Rehabilitation Sciences, Rady Faculty of Health Sciences, University of Manitoba, R106-771 McDermot Ave, Winnipeg, MB R3E 0T6 Canada; 5 Department of Community Health Sciences, Rady Faculty of Health Sciences, S113-750 Bannatyne Avenue, Winnipeg, MB R3E OW6 Canada

**Keywords:** Physiotherapy, Central intake, System reorganization, Referral, Wait list, Complex needs, Satisfaction

## Abstract

**Background:**

Children with complex needs can face barriers to system access and navigation related to their need for multiple services and healthcare providers. Central intake for pediatric rehabilitation was developed and implemented in 2008 in Winnipeg Manitoba Canada as a means to enhance service coordination and access for children and their families. This study evaluates the process and impact of implementing a central intake system, using pediatric physiotherapy as a case example.

**Methods:**

A mixed methods instrumental case study design was used. Interviews were completed with 9 individuals. Data was transcribed and analyzed for themes. Quantitative data (wait times, referral volume and caregiver satisfaction) was collected for children referred to physiotherapy with complex needs (*n* = 1399), and a comparison group of children referred for orthopedic concerns (*n* = 3901). Wait times were analyzed using the Kruskal-Wallis test, caregiver satisfaction was analyzed using Fisher exact test and change point modeling was applied to examine referral volume over the study period.

**Results:**

Interview participants described central intake implementation as creating more streamlined processes. Factors that facilitated successful implementation included 1) agreement among stakeholders, 2) hiring of a central intake coordinator, 3) a financial commitment from the government and 4) leadership at the individual and organization level. Mean (sd) wait times improved for children with complex needs (12.3(13.1) to 8.0(6.9) days from referral to contact with family, *p* < 0.0001; 29.8(17.9) to 24.3(17.0) days from referral to appointment, *p* < 0.0001) while referral volumes remained consistent. A small but significant increase in wait times was observed for the comparison group (9.6(8.6) to 10.1(6.6) days from referral to contact with family, *p* < 0.001; 20.4(14.3) to 22.1(13.1) days from referral to appointment, *p* < 0.0001), accompanied by an increasing referral volume for this group. Caregiver satisfaction remained high throughout the process (*p* = 0.48).

**Conclusions:**

Central intake implementation achieved the intended outcomes of streamlining processes and improving transparency and access to pediatric physiotherapy (i.e., decreasing wait times) for families of children with complex needs. Future research is needed to build on this single discipline case study approach to examine changes in wait times, therapy coordination and stakeholder satisfaction within the context of continuing improvements for pediatric therapy services within the province.

**Electronic supplementary material:**

The online version of this article (doi:10.1186/s12913-016-1700-3) contains supplementary material, which is available to authorized users.

## Background

The amount of time that a child waits to receive rehabilitation services is an issue of national and international concern [1], and families of children with complex needs may be particularly susceptible to long waiting periods. By definition, a child with complex needs requires services from multiple providers, often in multiple locations and under one or more sectors [2] including health, education and family services. Most often, these children live with neurodevelopmental impairment. Lack of coordination between service providers and restrictive policies about sharing confidential information between providers and across sectors [3] can contribute to inefficiencies and delays for families of children with complex needs.

Physiotherapy is one of the many services that a child with complex needs may require, and the importance of improved access to pediatric physiotherapy has long been recognized [4]. The evidence to guide effective system reorganization in rehabilitation and specifically in physiotherapy is limited [5]. Previous studies have examined service reorganization approaches in rehabilitation such as offering a reduced wait time for patients in exchange for a brief (30 min), rather than full (60 min) consultation with the therapist [1] to improve throughput. Others have increased the focus on group treatment or community consultation [5]. Camden and colleagues reported that although parents were generally satisfied with a system reorganization that included group treatment, they continued to report dissatisfaction and concern with wait times and access to therapy [5], suggesting that these might be key factors to address to enhance client experience. Our team was unable to find any reports that described the reorganization of referral and intake systems in pediatric rehabilitation as a means of addressing issues of poor service coordination and lengthy wait times.

Central intake has been used to address wait times in other areas such as mental health [6], arthritis care [7] and gastroenterology [8]. It “provides a single point of entry for patients requiring access to more specialized services tailored to their needs” [6]. Central intake, as a form of system reorganization alone or in combination with regularly occurring quality improvement initiatives, may be a promising approach to improve wait times for rehabilitation services for children with complex needs.

### Context of the current study

In the late 1990s, Specialized Services for Children and Youth (SSCY) was created as “…an initiative focused on the integration and where possible, co-location, of services for Manitoba children and youth with disabilities and special needs.” [9] The SSCY Intersectoral Working Group (IWG) brought together government stakeholders, service providers and families, and created a vision for an integrated service delivery system which included central intake as a key component [10, 11].

The Children’s Therapy Initiative (CTI) began in 2002 in an effort to improve the coordination of audiology, occupational therapy, physiotherapy, and speech-language pathology services for children in Manitoba [12]. Through SSCY and CTI, central intake was embarked upon to “increase accessibility, coordination and encourage more equitable service” [12].

Prior to central intake, pediatric rehabilitation services were provided to preschool children in Winnipeg by six different service providers, funded by two different provincial government departments (Health and Family Services). Each provider had their own mandate and wait list. Children in need of rehabilitation services were often referred to multiple providers based on diagnosis rather than need for therapy, and there was little communication among and between service providers. Consequently children could be waiting for therapy at multiple sites and in some cases, be receiving treatment from two or more therapists from the same profession (e.g., physiotherapists working for different service providers and using different service delivery models). Physiotherapy was one of the first disciplines to start using the central intake system.

### Purpose and objectives

The purpose of this study was to investigate the processes and impact of implementing a central intake system, using one discipline, pediatric physiotherapy as a case example. The primary objective was to explore what changes occurred as the result of central intake, the factors that influenced change and lessons learned. A secondary objective was to determine the impact of central intake implementation on outpatient wait times for physiotherapy at one site. Our final objective was to evaluate the impact of central intake implementation on caregiver satisfaction with physiotherapy services. We evaluated the impact of central intake implementation for families receiving therapy from the Child Health Physiotherapy team at Health Sciences Centre (HSC) in Winnipeg Manitoba. Health Sciences Centre Winnipeg is a major provider of pediatric services for the province of Manitoba, and is often the first point of contact for children with complex needs requiring rehabilitation. Ethical approval was obtained from the University of Manitoba Health Research Ethics Board, in accordance with the Declaration of Helsinki, prior to initiating the project.

## Methods

### Design

An instrumental single case study design was used to capture the perspectives of key actors in implementing central intake in its real world setting [13, 14]. Case studies use a specific case to understand an issue or phenomenon in greater depth [14]. They have value in both generating theory as well as generalizing understanding of existing theories and frameworks relative to specific contexts [15]. Both qualitative and quantitative methodology were used to enhance the internal validity of the case [13].

### Data sources

Qualitative data were collected from individual interviews with a purposefully selected sample of informants who were actively involved as practitioners or in leadership roles during the development of central intake. Interviews were conducted using a semi-structured interview guide (Additional file 1). Interviews lasted between 45 and 90 min. Topics of inquiry included exploring participants’ perspectives about key events in the development of central intake, changes to the referral processes pre- and post-central intake implementation and factors influencing change related to the development and implementation of central intake. All interviews were conducted by the same study team member (KM), a physiotherapist with 35 years’ experience in child health, to maintain consistency. Interviews were audio taped and transcribed verbatim.

The impact of central intake implementation on caregiver satisfaction with physiotherapy services, responsiveness and referral volume were assessed quantitatively by accessing electronic data that have been routinely collected as part of ongoing quality assessment within the physiotherapy department. Data collected from January 2006 to April 30, 2008 was defined as ‘pre-central intake implementation’ while data collected from May 1, 2008 to December 2012 was defined as ‘post-central intake implementation’. Although central intake was implemented primarily to coordinate outpatient referrals for pre-school children with complex needs (i.e., children with neurodevelopmental conditions), we also analyzed data from children and adolescents referred for orthopedic conditions during this time to assess for potential unintended impacts of central intake implementation on this clinical comparison group. Clinical data collection methods permitted the analysis of responsiveness and referral volume for the outpatient neurodevelopmental and orthopedic service areas separately, but satisfaction with outpatient therapy services could not be analyzed separately by service area. Practice changes that co-occurred during this time were not accounted for or analyzed within this study, and data from all age groups accessing physiotherapy services within the child health program during this time period were included.

### Measures

#### Responsiveness

Two measures of responsiveness were used; i) the time period from receipt of referral to time of first contact with patient/family (receipt to contact), as well as ii) time from receipt of referral to first appointment (receipt to appointment). By definition, the second time frame includes variability in the availability of therapy appointments, family availability and/or family scheduling preferences. In addition to analyzing responsiveness by service area (neurodevelopmental, orthopedic), analysis was also conducted by priority levels as defined by the site (Table 1). Children with complex needs would typically meet criteria listed within “neurodevelopmental priority 2”.

**Table 1:** Definitions of priority levels by service area (neurodevelopmental, orthopedic)

Physiotherapy Outpatient Service Area & Priority Level	Target for child to be seen by therapist	Description: Infant, child or youth referred due to the impairments listed below
Neurodevelopmental 1	3–5 working days	Acute illness/injury or acute exacerbation of existing problem; for example: brain injury, Guillain-Barré Syndrome, spinal cord injury, oncologic diagnosis, post-op neurosurgery
Neurodevelopmental 2	2–4 weeks	Under 2 years of age with neurological impairment; for example: abnormal muscle tone, delayed motor development Toe walking with neurological involvement
Neurodevelopmental 3	4–8 weeks	Older than 2 years of age with mild motor dysfunction Older than 2 years with neurological impairment or developmental delay, for whom no other resources exist
Orthopedic 1	1–3 working days	Surgery involving joint or muscle/tendon unit, especially hands Potential/actual reflex sympathetic dystrophy Intra-articular fracture, fracture dislocation Newly diagnosed arthritis/rheumatological condition Newborn brachial plexus
Orthopedic 2	1–2 weeks	Torticollis and/or plagiocephaly Musculoskeletal injury or pain: recent onset less than 3 months Decreased range of motion following fracture or joint injury
Orthopedic 3	3–4 weeks	Musculoskeletal injury or pain: long-standing (more than 3 months) Metatarsus adductus, intoeing, toewalking less than 4 years of age Spinal deformity, with brace

#### Referral volume

The number of referrals received by the pediatric physiotherapy department was tabulated for neurodevelopmental outpatient caseload and the orthopedic caseload separately, on a monthly basis from 2006 to 2012.

#### Client satisfaction

A satisfaction survey (non-validated) was conducted annually from 2006 to 2012 as part of ongoing quality of care monitoring. The survey was developed by physiotherapy managers and clinicians with the purpose of achieving a common metric of client (in this case, caregiver) satisfaction that could be assessed across multiple service areas of physiotherapy practice within the centre, on an annual basis. Paper surveys were distributed for a one-month period, and responses were compiled for analysis and reporting. The frequency and percentage of respondents who rated their satisfaction with physiotherapy as “very good” or “excellent” was calculated as an annual measure for quality reporting, and thus available to be used for analysis within the present study. Data was only available for service areas and priority levels combined.

### Analysis

#### Qualitative analysis

NVivo^©^ Version 10 software was used for data management and analysis. An initial coding scheme was developed from the key topics in the semi-structured interview guide. Descriptive and pattern codes and sub-codes were added to the coding scheme through line-by-line sequential review of the transcripts. Codes were grouped into themes and the relationships among the themes were interpreted. Memo writing during the analysis served to elaborate assumptions and identify similar and contrasting patterns in the data [16]. To maintain rigor during data analysis transcripts were checked for errors in transcription. Trustworthiness was promoted by maintaining field notes to document the interviewer’s perceptions and interpretations during each interview, and an audit trail to document the researcher’s decisions and actions made throughout the research process [17]. One member of the research team (KM) conducted the initial analysis of all transcripts. As a method of triangulation, a second member of the team (GR) read all transcripts and provided additional perspectives of the analysis and interpretation. Readily available historical documents (charters and presentations) [11] were also reviewed, and used to verify timelines and partners as needed.

#### Quantitative analysis

Assessment of data quality was undertaken using measures of completeness, temporal consistency, and accuracy, as per the data quality framework [18]. Responsiveness times are summarized by their means, standard deviations, and median values. The pre- and post-central intake distributions of responsiveness were formally compared via the Kruskal-Wallis test, owing to the highly skewed values and large presence of outliers. Secondary analyses compared responsiveness times separately for various service and priority codes, (e.g., neurodevelopmental, orthopedic; priorities 1–3). Wait times greater than 100 days were considered to be erroneous (verified by hand searching cases with wait times >100 days) and were removed from the analysis. *P*-values less 0.05 indicate the two periods significantly differ in their responsiveness times.

Monthly referrals were analyzed with a change-point regression model [19] that tests for a change in referral volume over time while accounting for any trends that may have been occurring prior to the central intake initiative. The model uses the data to estimate when the change was likeliest to occur, a more flexible and principled approach than assuming the effect on volume immediately coincided with the initiative. A significant change in slope taking place after the intervention indicates a change in referral volumes, or in their growth rates. The Durbin-Watson test indicated an absence of significant autocorrelation, and therefore was not necessary to take it into account. Pre- and post-central intake patient satisfaction levels were compared using the Fisher exact test, with responses being dichotomized as being either satisfied or dissatisfied. All analyses were performed using SAS version 9.3 (SAS Institute, Cary NC). The change-point model was implemented using PROC NLIN.

## Results

Interviews were conducted with nine participants from five sites, including individuals who provided direct care, were in leadership positions and/or coordinator positions. Five were physiotherapists; the non-physiotherapy participants held roles within the CTI and/or the Central Intake Steering Committee.

### Perspectives of impact: streamlining intake processes across sites

Participants talked about a system that was previously a “mess across a continuum” (P6) of physiotherapy services at various sites. Although they perceived that each site had logical processes, each worked as “a silo.” (P7). For families and referral sources the system was difficult to navigate with multiple points of access.

The complexity of the intake system created inequities among families. As one participant noted:

I think it was difficult and complicated. And (some) families phoned around lots of different places and got their names on lots of different lists and families who didn’t understand the system as well, could wait 18 months or 24 months and not see anybody. (P4)

Because each program and service had their own wait lists and there was little communication between sites, duplication of services often resulted:

For families, what we’ve found is that, um, they were often on multiple wait lists. They didn’t really know what list they were on or who they were waiting for. Um. And in some cases, because of that confusion, uh, they might be accessing physio in two different settings, working on the same goals and areas. (P1)

All participants identified positive changes after implementation of central intake. One participant described the overall transformation’s impact on parents this way:

We were in almost a mess, you know. Everything all over the place and everybody confused, you know. Not just families but all of us, you know. And, uh, and then to get to this point where it just seems more streamlined … more information, … more availability, …, and make sure that things are … being accomplished for families …. I just think it’s fantastic …. Big accomplishment. (P3).

Changes occurred in five major areas: reduced service duplication for children, more equitable wait times for service, increased communication among clinicians, clearer and more transparent processes for accessing services, and more accurate data about wait times. Table 2 summarizes illustrative participant comments about these changes. Participants believed that changes benefited clinicians, referral sources and most importantly, children and their families. Although some participants identified further improvements that could be made to the central intake process, in particular better data management systems, none identified ways in which the process had become worse. In addition to identifying the outcomes of the transition of individual intake systems for physiotherapy for six service providers funded under two different government departments (Health, Family Services) to a central intake system, this study also explored the influencers of change.

**Table 2:** Major themes illustrating the impact of central intake implementation

Theme	Participant quotes
Reduced service duplication	(P1) We no longer have unwarranted service duplication. (P8) We just don’t (have children)…on multiple lists.
More equitable wait times based on priority need	(P4) I think it’s working for families. And, I mean families still have to wait. So that’s, um, not good. But I think it’s an easier system to navigate. (P8) I think that’s been one of the benefits for the therapists too, right, is that they have a clearer sense of the priorities.
Increased communication among therapists	(P5) There’s a discussion that usually happens between the two therapists (at 2 different sites) so that you can kind of tweak out when that service is going to happen. (P7) I do know like just communication, uh, between therapists is better.
Clearer, simpler and more transparent processes for accessing the service in the right location	(P6) The central intake, I think it just paved the way for us to stream people to the right destination, the right location and make it easier for everybody to, uh, know who’s in the queue and to have even a way to track where people were. (P1) We have children going to the appropriate provider and being served based on their needs, not on diagnosis. (P3) One of the things is that one central point of entry, you know, for everybody. Like for the families, you know. For physicians. For, you know, uh, all of the support people in the community, you know, to come through one route, you know, is, uh, to me like fabulous. You know like that’s it right there in a nutshell, you know. (P4) And certainly some of the evaluative things that I’ve heard are just, um, particularly from the PT side …. there’s like hardly any complaints from families or from referring people. (P5) So it’s a better understanding I think from the families of what services are out there for physiotherapy at least. And, and that’s huge….There’s more satisfaction.
More accurate wait time data	(P7) Wait list under control, you know, like in terms of, um, knowing who’s who and who’s waiting and who’s got what and how long have they been waiting. (P1) We now know where every child is within the system. Nobody’s guessing anymore….central intake can certainly answer the question. (P4) I think the waiting list has been really cleaned up. Like I think it’s accurate for the first time.

### Factors that influenced change

Informants identified four themes related to primary facilitators of the change process. First, there was broad agreement across all stakeholders that there was a problem that needed to be fixed. Participants reported that families, frontline clinicians, managers and government funders had all expressed frustration with the existing system. One participant stated: “Everyone was so fed up with the system the way it was so that there was a willingness to … work at this collaboratively and see if we can’t come up with something different.” (P1) Second, stakeholders perceived that the hiring of a central intake coordinator who could bring together the multiple waitlists was a feasible and relatively low cost solution to the problem. Third, the government provided funding to facilitate better therapy services including the hiring of a central intake coordinator.

And we made lots of changes that weren’t money related as well. But I don’t think people would have sat around the table if there hadn’t been a little bit of money on it. (P4)

Finally, participants identified the important role of committed leadership in steering the change process. Leadership at the individual and organizational levels assisted in overcoming barriers to implementation including: negotiating processes that were consistent with privacy legislation, sharing infrastructure such as electronic data management systems and office space, and developing committee structures to facilitate communication amongst stakeholders.

These factors facilitated the change required to implement a central intake system. Participant narratives suggested that the process of developing and implementing central intake also provided an opportunity for practice innovation. The process of establishing central intake facilitated relationships and trust among stakeholders that could be sustained, created communication structures including committees and working groups, and facilitated agreement to examine and define practice across settings. The vision of central intake was to “ensure pre-school children are seen as quickly as possible, by the most suitable service provider in the most appropriate location” [20]. To actualize this vision each setting needed to be clear about physiotherapy practice and service delivery models in that setting, creating an opportunity to critically reflect on current practice.

### Quantitative results

The above section explored the processes and impact of central intake broadly across physiotherapy sites and providers. The following section illustrates the impact on a single site: HSC Winnipeg.

#### Responsiveness: receipt of physiotherapy referral to contact with family

The Child Health Physiotherapy team at HSC Winnipeg receives a wide range of referrals from acute isolated injuries to more complex injuries and conditions that require multidisciplinary and/or tertiary care (Table 1). Quantitative data were analyzed first with outpatient services areas and priority levels combined, and then separate.

With outpatient service areas and priority levels combined, there was no difference in the time from receipt of referral to contact with family (mean ± sd; 9.5 ± 11.1 d to 9.2 ± 7.7 days, NS) with the implementation of central intake. However, there were significant changes observed within the individual service areas. For the population of interest, i.e., children affected by neurodevelopmental conditions, there was an overall decrease in time to contact from 12.3 ± 14.1d to 9.0 ± 7.9 days (*p* = 0.003) when priority levels were combined. For those referred for orthopedic conditions, there was a small but significant increase in time to contact, from 8.3 ± 9.3d to 9.2 ± 7.6 days (*p* < 0.0001).

This was further examined by testing for differences in time to contact for each priority level within each of the two service areas (Table 3). For the neurodevelopmental group, there was a significant reduction in the time to contact for the priority 2 category (Table 3); i.e., those that could be considered to have complex needs. There was a small but significant increase in time to contact in the priority 2 category within the orthopedic service.

**Table 3:** Time (days) from receipt of physiotherapy referral to contact with family

Priority	Period	*n*	Mean (sd)	Median	*p*-value
Orthopedic & Neurodevelopmental Services Combined					
1	**Pre** ^**a**^	**207**	**3.9 (9.3)**	1	**0.003**
	**Post** ^**b**^	**491**	**1.8 (5.5)**	1	
2	Pre	758	10.5 (10.4)	8	0.56
	Post	3174	9.6 (6.7)	8	
3	Pre	62	16.7 (15.9)	13.5	0.06
	Post	608	12.2 (9.0)	10	
Neurodevelopmental Service Only					
1	Pre	33	11.2 (17.9)	5	0.16
	Post	22	9.5 (19.3)	0	
2	**Pre**	**263**	**12.3 (13.1)**	**9**	**<0.0001**
	**Post**	**814**	**8.0 (6.9)**	**7**	
3	Pre	9	18.6 (25.3)	13	0.64
	Post	258	11.8 (8.6)	9	
Orthopedic Service Only					
1	Pre	174	2.5 (5.6)	1	0.07
	Post	469	1.4 (3.5)	1	
** 2**	**Pre**	**495**	**9.6 (8.6)**	**8**	**<0.001**
	**Post**	**2360**	**10.1 (6.6)**	**9**	
3	Pre	53	16.4 (14.1)	14	0.10
	Post	350	12.4 (9.3)	10	

Bold *p*-values indicate a result that is statistically significant at α =0.05

^a^Pre: refers to the pre-central intake implementation period

^b^Post: refers to the post-central intake implementation period

#### Receipt of physiotherapy referral to appointment

Again, with service areas and priority levels combined, there was no change in time from receipt of referral to appointment (mean ± sd; 19.7 ± 19.0 d to 20.6 ± 17.6 days, NS). However, when examined by service area, time to appointment decreased with central intake implementation from 30.4 ± 21.2 d to 28.4 ± 20.6 days (*p* = 0.03) for those referred for neurodevelopmental conditions, while an increase was observed within the orthopedic service group (15.2 ± 15.9 to 17.8 ± 15.6 days, *p* < 0.0001). Further examination of wait times within service areas, categorized by priority suggests that these changes were again driven by changes experienced within the priority 2 category for both groups; a decreased wait time for those within the neurodevelopmental service and an increase for those within the orthopedic service (Table 4).

**Table 4:** Time (days) from receipt of physiotherapy referral to appointment

Priority	Period	*n*	Mean (sd)	Median	*p*-value
Orthopedic & Neurodevelopmental Services Combined					
1	Pre^a^	636	12.8 (19.6)	4	0.81
	Post^b^	1770	13.2 (20.2)	5	
2	Pre	838	23.7 (16.3)	20	0.60
	Post	3039	22.7 (14.3)	20	
3	Pre	75	32.5 (21.2)	29	0.60
	Post	597	30.2 (16.7)	26	
Neurodevelopmental Service Only					
1	Pre	163	30.7 (25.5)	27	0.11
	Post	384	34.8 (26.9)	30	
2	**Pre**	**289**	**29.8 (17.9)**	**28**	**<0.0001**
	**Post**	**811**	**24.3 (17.0)**	**21**	
3	Pre	12	37.3 (30.4)	21	0.87
	Post	255	31.7 (16.4)	28	
Orthopedic Service Only					
1	Pre	473	6.6 (12.1)	1	0.43
	Post	1385	7.3 (12.5)	1	
** 2**	**Pre**	**549**	**20.4 (14.3)**	**17**	**<0.0001**
	**Post**	**2228**	**22.1 (13.1)**	**20**	
3	Pre	63	31.6 (19.2)	32	0.25
	Post	342	29.1 (16.9)	26	

Bold *p*-values indicate a result that is statistically significant at α =0.05

^a^Pre: refers to the pre-central intake implementation period

^b^Post: refers to the post-central intake implementation period

#### Referral volume

**Fig. 1:**
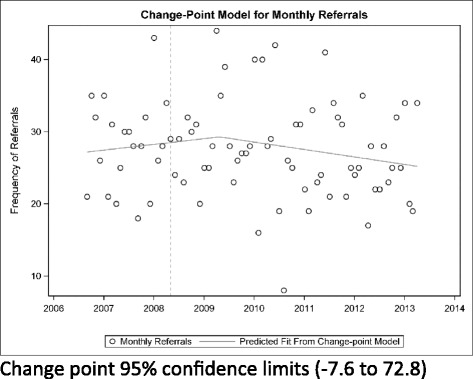
Change-point model for monthly referrals in the pediatric physiotherapy neurodevelopmental service (*p* = 0.30)

**Fig. 2:**
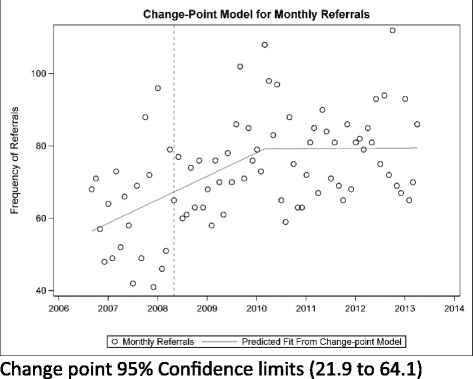
Change-point model for monthly referrals in the pediatric physiotherapy orthopedic service (*p* = 0.04)

Change point regression models indicate that there was no significant change in monthly referral volume in the neurodevelopmental outpatient service during the period from 2006 to 2012 (Fig. 1). In contrast, referral volume was increasing during central intake implementation for the orthopedic service; leveling off at a point approximately 2 years after implementation (Fig. 2).

#### Satisfaction

Patient satisfaction was high in both the pre- and post-central intake implementation periods. In the pre-implementation period, 96.6 % of respondents reported their satisfaction as very good or excellent, compared with 98 % in the post-implementation period (*p* = 0.48).

## Discussion

Central intake implementation for pediatric physiotherapy functioned to streamline processes across sites. System transformation led to more accurate, equitable and transparent wait times, better communication among therapists and reduced service duplication. The data that were available within the clinical systems confirmed these themes arising from stakeholder interviews. Children within the neurodevelopmental outpatient caseload at HSC Winnipeg were able to attend physiotherapy sooner after referral with central intake implementation; a change that appears to be driven specifically by shorter wait times for children in the priority 2 category. By definition (Table 1), these are the children who would be considered to have complex needs; thus, central intake worked to improve access to physiotherapy for a group of children who are at risk of being most disadvantaged by an uncoordinated system.

The findings from our qualitative data highlighted important facilitators of the change process. Many models and theories focus on change management within organizations. Our case study involved change across organizations within a system. However, our findings for system change highlighted important similarities between Kotter’s model of organizational change [21], and the perspectives of the informants in our study about the actions that resulted in a successful system change. Consistent with the first stage in Kotter’s model, there was a sense of urgency; most stakeholders agreed there was a problem that needed to be fixed. However, the impetus for bringing together a powerful coalition (stage 2) was a directive by the provincial government funder of pediatric rehabilitation services across the organizations accompanied by a small amount of funding contingent on working together to find a solution. This directive brought together leaders of independent organizations to work as a team. The team was able to create a vision of central intake as one initial solution to the problem (stage 3), communicate the vision (stage 4), and bring in others within organizations, including a central intake coordinator, to act on the vision (stage 5). The perceived success of the central intake process served as a short term win (stage 6) to catalyze other potential practice innovations and consolidate improvements (stage 7). Institutionalizing new approaches (stage 8) is anticipated to occur over time [21]. Interestingly, the above stages and perspectives align well with the “5 Rs of Reorganization” which were developed and used by Phoenix and colleagues in a 2016 case report detailing change in an Ontario pediatric rehabilitation setting [22]. Briefly, with the 5Rs Phoenix et al. provide the following 5 steps to guide planning for service delivery change in pediatric rehabilitation: 1. Recognize need for change, 2. Reallocate resources, 3. Review reality, 4. Reconstruct reality and 5. Report results [22].

This is the first report that we are aware of to detail and quantify the process of central intake implementation in pediatric physiotherapy. Multiple facilitators were identified that can help inform other jurisdictions wishing to implement central intake or a related system overhaul. Agreement among stakeholders that a change was needed, the hiring of a central coordinator, financial commitment from government and importantly, strong leadership at multiple levels resulted in the successful development and implementation of a new intake system. Similarly, a perception of responding to families’ needs, project funding and leadership were among the facilitators identified within a study of service model reorganization in pediatric rehabilitation by Camden and colleagues [23].

Of importance in this retrospective study was the mixed methods approach of including the perceptions of change from leaders of the change process and the routine and consistent collection of data related to the impact of the change on wait times and satisfaction metrics. Including both types of data allowed us to examine both the extent and quality of the change. We recommend that others seeking to evaluate system change plan prospectively for the collection of both quantitative and qualitative data throughout the process, beginning in the pre-implementation phase.

Limitations of this work include that we evaluated only a case example of central intake implementation in one discipline, pediatric physiotherapy, while this was a provincial system change that involved and affected other disciplines and sites. Thus, our results alone do not capture the full impact of central intake, even for physiotherapy where we expect that the impact was even larger if all sites were considered. Due to the retrospective nature of the study however, we were unable to ensure alignment of data capture systems and metrics to evaluate the effects of central intake on all providers simultaneously. Ongoing efforts through the SSCY initiative and its committees will build on the data presented here to explore changing trends in wait times, therapy coordination, stakeholder satisfaction and outcomes across service providers, and within the context of continued improvements such as service provider co-location. Another limitation of this study includes a wide variability in the quantitative data, as evidenced by large standard deviations. We attempted to clean the data by selecting a cut-off of 100 days wait time based on clinical impression that this was an excessive wait at this site. This was confirmed by hand searching cases with >100 day wait, which revealed data entry errors, and/or cases where a referral was originally intended for another service (e.g., occupational therapy) and transferred to physiotherapy using the original referral date. Overall, a small percentage of data were excluded for exceeding this threshold (time to contact: 0.72 %; time to appointment: 2.1 % of data were excluded). Despite the remaining variability in the data we did observe a significant reduction in the wait time for children in the neurodevelopmental priority 2 group.

We were interested in the small but significant increase in wait times for children referred to the outpatient orthopedic service (approximately 1–1.5 days). While central intake was not designed to change wait time for this caseload, we performed the analyses for this group to be able to detect unintended consequences of a system change that altered services for another outpatient service at the same site. We were unable to determine from the qualitative data collected for this study if the increase in wait was related to central intake. It is important to note that change point analysis indicated an increase in the number of monthly referrals to the orthopedic service over a 4 year period spanning central intake implementation; this could have lengthened wait times. An alternate explanation is that the small increase is a false positive, resulting from a large sample size.

## Conclusion

The key functions of the central intake program were to “i) provide an integrated intake system that supports efficient collection of information, provides accurate and relevant information to clients, service providers and referral sources, and minimizes client transitions between service providers; ii) maintain a centralized client registry for children accessing/requiring therapy services, and iii) maintain a centralized wait list for services that supports efficient utilization of agency resources, eliminates service duplication, and supports timely and equitable access to services” [24]. This study demonstrates that central intake implementation significantly improved access to pediatric physiotherapy at HSC Winnipeg for children with complex needs. Central intake contributed to transparent and equitable access to services, and as a process, was facilitated by commitment to the objective and strong leadership at multiple levels. There was evidence that having stakeholders and service providers together at a central table to strategize around central intake served as a facilitator for co-occurring practice change. This is a finding that will be more thoroughly evaluated in future research.

## Additional file

Additional file 1: Semi-structured interview guide. (DOC 24 kb)

## Abbreviations

CTI: Children’s Therapy Initiative
HSC: Health Sciences Centre
IWG: Intersectoral Working Group
SSCY: Specialized Services for Children and Youth

## References

[1] Clow D, Mustafa A, Snollar J, Wood N, Reid J, Sinden S (2002). Reducing waiting times associated with integrated child health service. J R Soc Promot Health.

[2] Burnside L. Youth in care with complex needs. Special Report for the Office of the Children’s Advocate. 2012. http://www.childrensadvocate.mb.ca/wp-content/uploads/Youth-with-Complex-Needs-Report-final.pdf. Accessed 12 Feb 2016.

[3] Miller AR, Armstrong RW, Masse LC, Klassen AF, Shen J, O’Donnell ME (2008). Waiting for child developmental and rehabilitation services: an overview of issues and needs. Dev Med Child Neurol.

[4] Martin T (1989). Message from the Chair: Access and advocacy. Pediatr Phys Ther.

[5] Camden C, Swaine B, Tetreault S, Brodeur MM (2010). Reorganizing pediatric rehabilitation services to improve accessibility: do we sacrifice quality?. BMC Health Serv Res.

[6] Cloutier P, Cappelli M, Glennie JE, Charron G, Thatte S (2010). Child and youth mental health service referrals: physicians’ knowledge of mental health services and perceptions of a centralized intake model. Healthc Policy.

[7] Barber CE, Patel JN, Woodhouse L, Smith C, Weiss S, Homik J (2015). Development of key performance indicators to evaluate centralized intake for patients with osteoarthritis and rheumatoid arthritis. Arthritis Res Ther.

[8] Novak K, Veldhuyzen Van Zanten S, Pendharkar SR (2013). Improving access in gastroenterology: the single point of entry model for referrals. Can J Gastroenterol.

[9] Specialized Services for Children and Youth. 2016. http://www.sscy.ca. Accessed 4 Feb 2016.

[10] Specialized Services for Children and Youth: About SSCY Network. 2016. http://sscy.ca/about-sscy/about-sscy. Accessed 4 Aug 2016.

[11] Susinski C. SSCY From Principles to Action. Presented at Quality Improvement Forum 2013: Inspiring Improvement. Vancouver. http://qualityforum.ca/qf2013/wp-content/uploads/2013/03/PFCC-Breakout-2-Cheryl-Susinkski1.pdf. Accessed 4 Aug 2016.

[12] Government of Manitoba: Student Services/Special Education – Intersectoral Initiatives, Children’s Therapy Initiative. www.edu.gov.mb.ca/k12/specedu/intersectoral/cti/#. Accessed 4 Feb 2016.

[13] Crowe S, Cresswell K, Robertson A, Huby G, Avery A, Sheikh A (2011). The case study approach. BMC Med Res Methodol.

[14] Stake R, Denzin NK, Lincoln YS (2005). Qualitative case studies. The Sage handbook of qualitative research.

[15] Flyyberg B, Denzin NK, Lincoln YS (2011). Case Study. The SAGE handbook of qualitative research.

[16] Miles M, Huberman AM, Saldana J (2014). Qualitative data analysis.

[17] Creswell JW, Miller D (2000). Determining validity in qualitative inquiry. Theory Pract.

[18] Lix L, Smith M, Azimaee M, Dahl M, Nicol P, Burchill C (2012). A Systematic Investigation of Manitoba’s Provincial Laboratory Data.

[19] Julious SA (2001). Inference and estimation in a changepoint regression problem. The Statistician.

[20] CTI Winnipeg-Central Intake Working Groups Project Charter. Version October 22 2012.

[21] Kotter J. Leading change: Why transformation efforts fail. Brighton: Harvard Business Review; 2007.

[22] Phoenix M, Rosenbaum P, Watson D, Camden C (2016). The “5Rs of Reorganization”: A Case Report on Service Delivery Reorganization within a Pediatric Rehabilitation Organization. Phys Occup Ther Pediatr.

[23] Camden C, Swaine B, Tetreault S, Carriere M (2011). Going beyond the identification of change facilitators to effectively implement a new model of services: lessons learned from a case example in paediatric rehabilitation. Dev Neurorehabil.

[24] Specialized Services for Children and Youth. WRHA Centralized Intake Service Announcement. 2008. http://www.wrha.mb.ca/professionals/familyphysicians/files/News_CITherapy_080923.pdf. Accessed 4 Feb 2016.

