# Supplementary material for: Central intake to improve access to physiotherapy for children with complex needs: a mixed methods case report
Source: BMC Health Serv Res. 2016 Aug 31;16(1):455. doi: 10.1186/s12913-016-1700-3 (PMC5006453; doi:10.1186/s12913-016-1700-3)
Supplement: Additional file 1: — Semi-structured interview guide. (DOC 24 kb) [file 12913_2016_1700_MOESM1_ESM.doc]

**Interview Template**

Date of interview:

Participant name:

Participant position:

Participant position at time of central intake development: (if different than above)

1. When did you become involved in the central intake project?
2. What was your primary role in the project?
3. What were other roles that you held / tasks that you were responsible for that were relevant to this project?
4. What is your recall / perception of what the referral / intake process was like before central intake?
   1. For the families?
   2. For the therapists?
   3. For the other referring professionals? (e.g. physicians)
5. Can you guide me through some of the major milestones of the project from your point of view?
   1. *Interviewer to prompt participant to provide a chronological narrative of the project development and implementation; requesting more details as necessary*
6. What were some of the major barriers that you recall?
   1. From the list that you provided, which would you choose as the top 3?
7. What were some of the major facilitators of this project?
   1. From the list that you provided, which would you choose as the top 3?
8. What are the most important changes that you have seen since common intake was put into place (in 2008)?
   1. What do you feel is the major contributor to these changes?
9. Based on what you have learned from this process, what would you do differently if you had to do it all over again? And/or Are there any ‘words of wisdom” you would share with others embarking on a similar project?
10. Is there anything else that you would like to add?
11. Can I contact you for any clarification about this interview?
